# Long non-coding RNA MIAT acts as a biomarker in diabetic retinopathy by absorbing *miR-29b* and regulating cell apoptosis

**DOI:** 10.1042/BSR20170036

**Published:** 2017-04-28

**Authors:** Jiayu Zhang, Maochong Chen, Jiawei Chen, Sisi Lin, Daqiu Cai, Chengwei Chen, Zhenguo Chen

**Affiliations:** Department of Ophthalmology, The Third Affiliated Hospital of Wenzhou Medical University, Zhejiang Ruian 325200, China

**Keywords:** cell apoptosis, diabetic retinopathy, high glucose, MIAT, miR-29b

## Abstract

Diabetic retinopathy (DR) is a complication of diabetes mellitus (DM) and is the leading cause of vision loss globally. However, the pathogenic mechanism and clinical therapy still needs further improvement. The biologic significance of myocardial infarction associated transcript (MIAT) in DR remains unknown. Here, we aim to explore the mechanism between MIAT and DR, which is essential for RD. Streptozotocin (STZ) was used to induce DM mice and high glucose was used to stimulate cells. ChIP was used to detect the binding activity between nuclear factor κB (NF-κB) and the promoter of the MIAT gene, luciferase activity assay was used to detect the target-specific selectivity between *miR-29b* and MIAT. The expressions of MIAT and p-p65 were increased in STZ-induced DM mice and high glucose stimulated rat retinal Müller cells (rMC-1) cells. ChIP results revealed that high glucose promoted the binding activity between NF-κB and MIAT, while Bay11-7082 acted as an inhibitor for NF-κB that suppressed the binding activity. *miR-29b* controled MIAT to regulate its expression and MIAT overexpression suppressed *miR-29b*, but promoted Sp1. High glucose stimulation increased the cell apoptosis and decreased the cell activity, while MIAT suppression reversed the effect induced by high glucose, however, *miR-29b* knockdown reversed the effects induced by MIAT suppression. Our results provided evidence that the mechanism of cell apoptosis in DR might be associated with the regulation of MIAT, however, *miR-29b* acted as a biomarker that was regulated by MIAT and further regulated cell apoptosis in DR.

## Introduction

Diabetes mellitus (DM) is a complex metabolic disorder and remains a disease with high number of incidences worldwide, especially in the developed countries [1]. DM is commonly derived from the defects in insulin secretion or insulin action or both of them and the chronic DM will induce the damage or dysfunction of several organs, such as heart, eyes, nerves, as well as kidney and blood vessels [2,3]. Indeed, about half of the DM patients are suffering from several complications, which bring great pain for the patients and the family. Diabetic retinopathy (DR) is one of the most important complications in DM, which is afflicting approximately 20% of adult diabetic patients and is also a leading cause of vision loss globally [4]. Although an improvement has been made in the DR therapy in the recent years, the prognosis remains poor [5]. Thus, exploring the potential mechanism underlying DM is essential for the clinical therapy.

Long non-coding RNA is a class of non-coding RNA with the length of more than 200 nts, but without the function of protein-coding capacity [6,7]. Studies have demonstrated that many LncRNAs play an important role in regulating gene expression in diverse biological processes or pathological mechanisms [8,9]. Increasing evidence supported that LncRNA acted as a diagnostic marker or therapeutic target in diseases. For example, LncRNA H19 acted as a carcinogenic gene and was involved in gastric cancer [10], colorectal cancer [11] as well as glioma cells [12]. Overexpression of HOTAIR transcript is associated with colorectal cancer [13], breast cancer [14] and hepatocellular cancer [15]. The BACE1AS has been reported to play a vital role in the aetiology of Alzheimer’s disease (AD) [16]. The Gas5 transcript was linked with the immune system [17]. Although the research of lncRNA in endocrine disease remains limited, the important roles of several genes in metabolism and endocrine system have been reported, such as PTEN-induced gene *PINK1*, which was associated with diabetic status [18]. The FADS was found to be regulated by the dietary fat content [19]. LncRNA myocardial infarction associated transcript (MIAT) is predominantly expressed in the heart and the brain tissue [9,20]. Researchers showed that the abnormal expression of MIAT was involved in the cell proliferation, apoptosis and migration in many diseases, such as myocardial infarction [21], microvascular dysfunction [9] and diabetes [22]. However, whether this LncRNA MIAT has a regulative effect on cell apoptosis in DR is still unknown.

miRNA is a class of non-coding RNAs with the length of approximately 22 nts and always function in post-transcriptional processes [23]. It has been proposed that miRNA has crucially regulated diverse biological processes of many human diseases [24], and studies have reported that miRNA is quite promising in defining molecular mechanisms in diseases, such as cancers [25], neurodegenerative diseases [26], cerebrovascular diseases [27], neurological diseases [28] and endocrine disorders [29]. In the recent years, plenty of miRNAs have been reported to mediated the onset and development of diabetes-related disorders. *miR-29b* belongs to the *miR-29* family, which acts as a tumour suppressor in many tumour researches. Study has reported that *miR-29b* negatively regulated osteoblast differentiation [30]. At the same time, *miR-29b* was differentially expressed in DM [31], however, whether *miR-29b* regulation plays an important role in DR remains unclear.

To date, increasing evidence has supported that a number of LncRNA harboured internally encode miRNA and acquire function by acting as the precursor to miRNA and then become capable of regulatory function. Plenty of studies confirmed that the oncogenic mechanisms of LncRNA-regulated diseases were always integrated by miRNA associations or LncRNA–miRNA interactions. Thus, in the present study, we investigate LncRNA MIAT function in DR by harbouring * miR-29b* and the goal was to explore the molecular pathways underlying DR.

## Materials and methods

### DM mouse model establishment

Twenty male Sprague–Dawley (SD) mouse 4–6 weeks old were purchased from Shanghai Bioray Laboratories lnc. The study was permitted by the Animal Care and Use Committee. All mice were randomly divided into two groups and housed in the same atmosphere with adequate food and water. DM mice were induced by intraperitoneal injection of streptozotocin (STZ, 60 mg/kg dissolved in 0.1 mol/l citrate buffer), control mice were established by intraperitoneal injection of citrate buffer (0.1 mol/l). Blood glucose was detected 72 h after the injection, the glucose concentration above 16.7 mM was considered as successfully established. The mice were killed and the Müller cells were isolated immediately after 72 h of injection.

### Cells isolation and culture

Müller cells were isolated from normal mice or STZ-induced mice. Briefly, the tissues were ground and dissolved by lysate and then centrifuged for collecting Müller cells, cells were washed and diluted by RPMI 1640 medium. Rat retinal Müller cells (rMC-1, obtained from EK-Bioscience, Biotechnology Co., Ltd. Shanghai Enzyme Research) and Müller cells were cultured in RPMI 1640 medium with 10% FBS at 37°C with 5% CO_2_ in a 24-well plate.

### Real-time PCR

Total RNA was extracted from Müller cells or rMC-1 by using TRIzol reagent (Invitrogen) according to its manufacturer’s instructions. RNA quality was measured by a spectrophotometer (Thermo Fisher). cDNA was synthesized by using 1 μg RNA and a commercially available kit (iScript™) according to the manufacturer’s instructions. Real-time PCR was performed using the instrument ABI 7000 PCR (Applied Biosystems, Japan). The relative amount of mRNA was calculated using 2^−ΔΔ*C*^_t_ method. Gene expression was normalized by β-actin. All data were obtained from three individual experiments. The primers used in the present study were synthesized from Suzhou GeneWiz Technologies Co., Ltd. (Suzhou, China).

### Western blot

To assess the protein expression of p-p65 and SP1, Western blot assay was used. Briefly, Müller cells and rMC-1 were isolated and lysed in RIPA lysis buffer, the protein was collected by centrifugation (GT10-1) and quantified by BCA assay kit (Beyotime). Immunoblotting assay was carried out on an SDS/PAGE (12% gel) to separate protein extracts. The membrane was incubated with anti-p-p65 or anti-SP1 (1:500, Sigma) antibodies as well as 5% milk solution of TBS buffer at 4°C for 24 h. Then, secondary antibodies were incubated with membrane for another 2 h. The bands were observed by ECL method, β-actin acted as the internal control.

### Cell stimulation

rMC-1 was stimulated by high glucose (25 mM) or normal glucose (5.5 mM). After 24, 48, 72 and 96 h, stimulated cells were collected for the following experiments.

### ChIP analysis

rMC-1 cells were fixed with formaldehyde, quenched with glycine and washed with cold PBS. Cells were then lysed on ice and chromatin was sheared. A centrifuge was used to clear the cell lysate, G magnetic dynabeads (Invitrogen) and target antibody were mixed with the cells and cultured in a 96-well plate at 4°C for one night. Then cells were washed and eluted in elution buffer, and the elution was reverse cross-linked and treated sequentially with RNaseA and proteinase K. Solid phase reversible immobilization (SPRI) was used in a 96-well plate to reverse cross-linked samples. Supernatants were separated and beads were washed, DNA was eluted in 40 μl EB buffer. For the library construction, a general SPRI clean-up containing addition of buffer with 2.5 mM NaCl and 20% PEG to the DNA reaction products was performed.

### Cell transfection

rMC-1 cells were cultured in a 96-well plate for 24 h,* miR-29b* inhibitor, si-MIAT, Ad (adenovirus)-MIAT (Ad-MIAT) or their negative control (NC), Ad-carrying GFP (Ad-GFP) transfected the cells by Lipofectamine 2000 reagent (Invitrogen) according to the manufacturer’s instructions. After 24 h, the transfection efficiency was measured by real-time PCR according to the manufacturer’s instructions. The *miR-29b* inhibitor, si-MIAT and NC were synthesized by Shanghai Yingjun Co., Ltd. (China).

### Cell viability

Cell viability was measured by a Cell Proliferation and Cytotoxicity Reagent Kit (MTT) (Roche Applied Science). The rMC-1 cells in the logarithmic phase were used in the experiment and cultured at 37°C with 5% CO_2_ on a 96-well plate, the cells were stimulated by high glucose and transfected with si-MIAT, si-MIAT and *miR-29b* inhibitor. After 24 h, cell viability was measured according to the manufacturer’s instructions on the MTT kit. Briefly, cells were incubated with MTT for 4 h, then the formazan crystals were visualized by a microscope at OD =570 nm. All experiments were performed for three l times.

### Cell apoptosis

rMC-1 cells transfected with si-MIAT, si-MIAT and *miR-29b* inhibitor were cultured at 37°C with 5% CO_2_ on a 96-well plate for 48 h, and then harvested and stained with propidium iodide (PI) (Sigma) for 30 min. The FITC-Annexin V Apoptosis Detection Kit (Biosciences, U.S.A.) based on the double staining with FITC-Annexin V and PI was used to detect the cell apoptosis level. A flow cytometry (FACScan) was used to analyse the apoptotic cells.

### Statistical analysis

All data were presented as means ± S.D. SPSS 18.0 was used for data analysis. Statistical differences were carried out by using one-way ANOVA. *P༜0.05 was considered as statistically significant difference.

## Results

### Overexpression of MIAT and p-p65 in Müller cells with STZ injection

**Figure 1:**
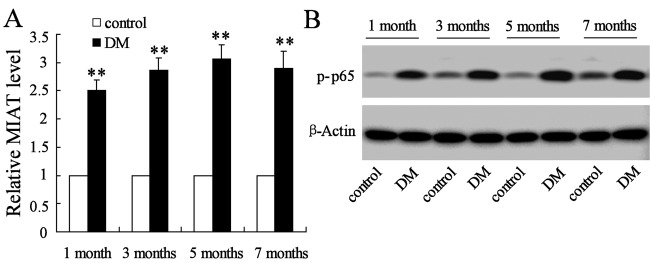
The expression level of MIAT and p-p65 in Müller cells (**A**) STZ supplementation significantly increased the expression level of MIAT than citrate buffer supplementation in mice. (**B**) The expression level of p-p65 was significantly increased in STZ-induced DM mice compared with citrate buffer-induced control. **P<0.01 compared with control, β-actin served as the internal control.

The Müller cells were isolated from STZ-induced DM mice or citrate buffer injected mice (control) after mice were injected with STZ or citrate buffer for 1, 3, 5 and 7 month.s Results demonstrated that the expressions of *MIAT* mRNA in STZ-induced DM mice were 2.5-, 2.8-, 3.1- and 2.9-fold and that of control in 1, 3, 5 and 7 months respectively (Figure 1A). Moreover, the protein expression of p-p65 was also significantly increased in the DM mice compared with the control (Figure 1B). The results indicated that MIAT and p-p65 were significantly up-regulated in DM mice.

### High glucose supplementation promoted the expression of MIAT and p-p65

**Figure 2:**
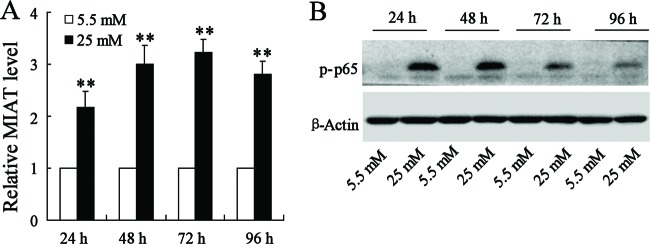
The effects of glucose concentration on the expression of MIAT and p-p65 Comparing with the normal glucose supplementation, the rMC-1 cells stimulated by high glucose contributed to the elevation of MIAT expression (**A**) and p-p65 expression (**B**). **P<0.01 compared with normal glucose supplementation, β-actin served as the internal control.

We examined the expression of MIAT and p-p65 by stimulation of rMC-1 cells with normal glucose or high glucose. After the stimulation for 24, 48, 72 and 96 h, the expression of MIAT and p-p65 were detected. As a result, *MIAT* mRNA levels in rMC-1 cells with high glucose stimulation were 2.2-, 3.0-, 3.3- and 2.8-fold and that of control in 24, 48, 72 and 96 h respectively (Figure 2A). Additionally, the protein expression of p-p65 was also significantly increased in high glucose stimulated rMC-1 cells rather than rMC-1 cells with normal glucose stimulation (Figure 2B).

### Effect of Bay11-7082 and high glucose on the binding activity of nuclear factor κB and MIAT

**Figure 3:**
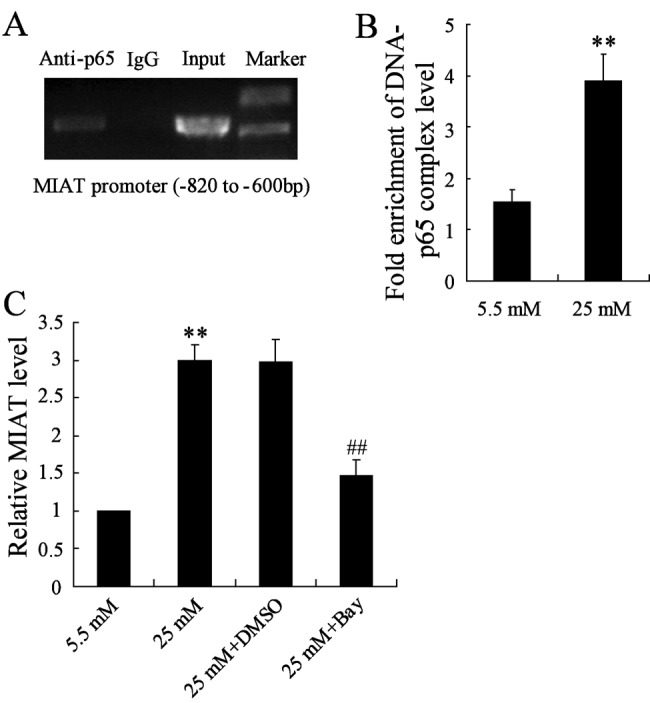
The relationship of NF-κB and MIAT (**A**) NF-κB directly binds to MIAT promoter. (**B**) High glucose promoted the binding activity of NF-κB and MIAT promoter in rMC-1 cells. (**C**) rMC-1 cells pretreated with Bay11-7082 and then stimulated by high glucose, the expression of MIAT was significantly decreased. **P<0.01 compared with normal glucose, ^##^*P*<0.01 compared with high glucose + DMSO.

To detect the binding activity of MIAT with its regulatory factor, we introduced a nuclear factor κB (NF-κB)-specific monoclonal antibody to chromatin immunoprecipitate DNA cross-linked to NF-κB-tagged proteins, and measured the enrichment of specific DNA sequences using real-time PCR after cells were stimulated by high glucose. Our ChIP assay demonstrated that NF-κB selectively binds to MIAT promoter (Figure 3A). Moreover, when rMC-1 cells were stimulated by high glucose, the binding activation was significantly increased compared with the normal glucose stimulation (Figure 3B). However, when rMC-1 cells were pretreated by Bay11-7082 (2.5 mM) for 2 h, and then stimulated by high glucose, the relative MIAT level was significantly decreased compared with the treatment of high glucose only (Figure 3C).

### Effects of si-MIAT on rMC-1 cells

**Figure 4:**
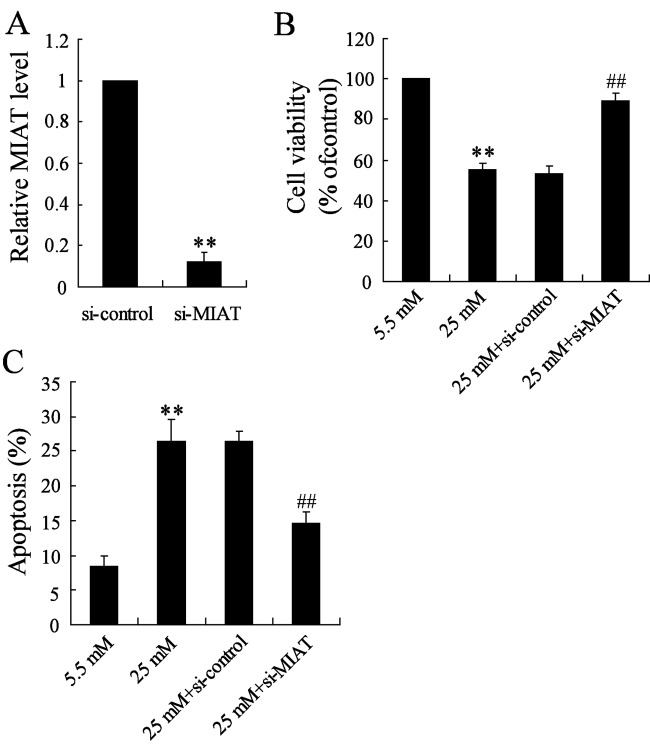
The effects of MIAT knockdown on cell viability and apoptosis in high glucose-induced rMC-1 cells (**A**) The transfected efficiency was significantly decreased. (**B**) MIAT knockdown significantly reversed the decrease in cell viability induced by high glucose. (**C**) MIAT knockdown significantly reversed the increase in cell apoptosis induced by high glucose. **P<0.01 compared with normal glucose, ^##^*P*<0.01 compared with high glucose + si-control.

In order to demonstrate the effects of MIAT suppression on rMC-1 cells, the si-MIAT was constructed and transfected into rMC-1 cells and then stimulated by high glucose for 96 h. The transfection efficiency of MIAT was detected; the results revealed that MIAT expression was significantly decreased compared with control (Figure 4A). Then we found that MIAT suppression reversed the significant decrease in cell survival rate induced by high glucose (Figure 4B). At the same time, MIAT suppression also reversed the increased rate of cell apoptosis induced by high glucose (Figure 4C).

### MIAT suppression increased the expression of* miR-29b* and SP1

**Figure 5:**
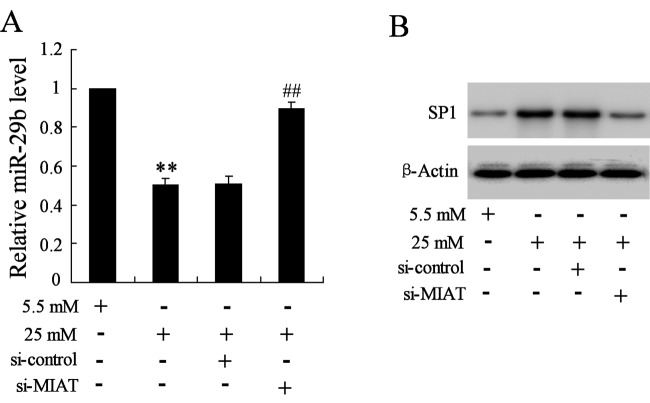
The effects of MIAT suppression on the expression of *miR-29* and Sp1 in high glucose-induced rMC-1 cells (**A**) MIAT suppression significantly reversed the decreased expression of *miR-29* induced by high glucose. (**B**) MIAT suppression significantly reversed the increase expression of Sp1 induced by high glucose. **P<0.01 compared with normal glucose, ^##^*P*<0.01 compared with high glucose + si-control.

In order to explore the potential mechanism between MIAT and cell apoptosis induced by high glucose, *miR-29b* was selected for further exploration. The real-time PCR reflected that when cells were pretreated with si-MIAT and then stimulated by high glucose, the expression of *miR-29b* was significantly increased than that treated by high glucose only (Figure 5A). While MIAT suppression also reversed the increase expression of Sp1 induced by high glucose (Figure 5B).

### MIAT targeted* miR-29b* to regulate its expression

**Figure 6:**
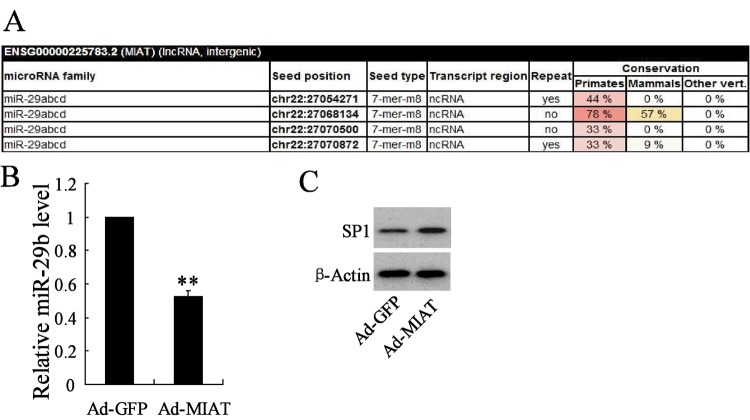
*miR-29b* targets MIAT to regulate its expression (**A**) TargetScan database predicted that *miR-29b* has highly conserved target sequence with 3′-UTR of MIAT. MIAT overexpression dramatically decreased the expression of *miR-29b* (**B**), while increased the expression of Sp1 in rMC-1 cells (**C**). **P<0.01 compared with Ad-GFP, β-actin served as the internal control.

We explored the relationship of *miR-29b* and MIAT. TargetScan database was used for the online prediction and the results revealed that *miR-29b* have highly conserved target sequence with MIAT (Figure 6A), the results indicated that MIAT could regulate *miR-29b* expression. In order to verify it, Ad-MIAT was constructed and transfected to rMC-1 cells and the expression of *miR-29b* and its downstream gene *SP1* was detected. Results revealed that MIAT overexpression significantly decreased the expression of *miR-29b* (Figure 6B), but increased the expression of SP1 (Figure 6C).

### Interaction of MIAT, *miR-29b* and high glucose on cell survival and apoptosis

**Figure 7:**
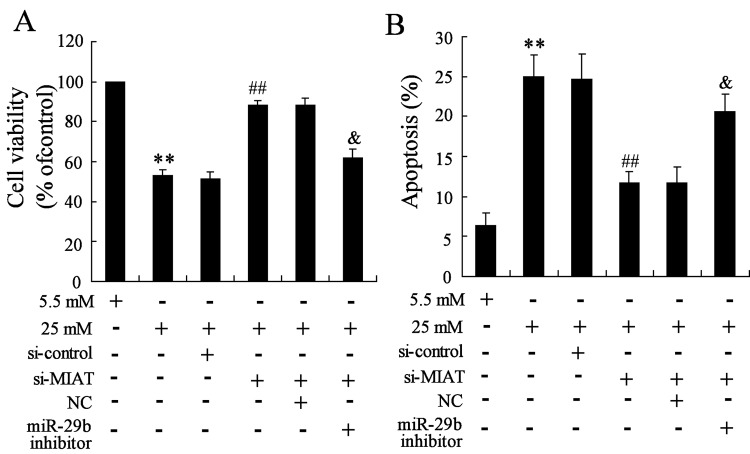
Interaction of MIAT and *miR-29b* on high glucose induced rMC-1 cells (**A**) MIAT suppression significantly reversed the decrease in cell viability induced by high glucose, while *miR-29b* knockdown significantly reversed the effect induced by MIAT suppression. (**B**) MIAT suppression significantly reversed the increase in cell apoptosis induced by high glucose, however, *miR-29b* knockdown significantly reversed the effect induced by MIAT suppression. **P<0.01 compared with normal glucose, ^##^*P*<0.01 compared with high glucose + si-control, ^&^*P*<0.01 compared with high glucose + si-MIAT + NC.

To identify the effects of MIAT, *miR-29b* on high glucose induced cell survival and apoptosis. rMC-1 cells were transfected with si-MIAT and *miR-29b* inhibitor, then high glucose was used to stimulate the cells. Results revealed that cell viability was significantly decreased and cell apoptosis was obviously increased by high glucose treatment, then MIAT suppression reversed the effects induced by high glucose, however, *miR-29b* knockdown significantly reversed the effects induced by MIAT suppression (Figure 7A,B).

## Discussion

STZ is synthesized by *Streptomyces achromogenes* and is widely used to induce diabetes. Previous studies reported that STZ leads to hyperglycaemia in mice, which is similar to diabetic person [32,33]. Thus, STZ-induced mice were always used for diabetic mice contribution. In the present study, STZ was intraperitoneally injected in mice for DM mice establishment, and the successfully established mice were used for the following experiments.

NF-κB is a heterodimer that comprised p65 and p50. NF-κB-p65 is always located in the cytoplasm, however, when the body is invaded by a disease, p-p65 is phosphorylated and then transferred into nucleus, the activated p-p65 acts as a transcription factor that regulates gene expression in nucleus [34]. According to the present study, the expression of p-p65 was significantly increased in DM mice and high glucose induced rCM-1 cells, indicating that the activity of NF-κB was increased in DM mice high glucose stimulated rCM-1 cells. Moreover, results revealed that NF-κB is directly bound with MIAT, indicating NF-κB targeted regulating the expression of MIAT. Then rCM-1 cells pretreated with Bay11-7082 significantly decreased the binding activation between NF-κB and MIAT than that induced by high glucose, indicating that Bay11-7082 just acted as an inhibitor that depressed the expression of MIAT.

Previous study investigated that Sp1 expression was directly targeted by *miR-29b*, which was bound to *miR-29b* promoter and repressed the expression of *miR-29b* [35 ]. At the same time, *miR-29b* inhibited the transcription of Sp1 and then up-regulated its own transcription [36]. From the present study, our results revealed that the expression of Sp1 was significantly increased in high glucose induced rMC-1 cells than MIAT directly targeted *miR-29b* expression, and MIAT suppression significantly reversed the low expression of *miR-29b* and high expression of Sp1 induced by high glucose. The results indicated that MIAT capable of this function might be through harbouring of *miR-29b* and then regulating the expression of *miR-29b* and Sp1.

DR is characterized by vascular lesions and macular oedema, which was accompanied by the insidious degeneration of vascular and neurons [37], however, the degenerative changes always accompanied with cell apoptosis . In this article, high glucose stimulation significantly increased cell apoptosis of rCM-1, which was in accordance with the previous study that showed cell apoptosis was in retinal diabetes [38], while the underlying mechanism was still unclear. In the present study, high glucose stimulation promoted cell apoptosis, then MIAT suppression reversed the high apoptosis induced by high glucose, indicating that MIAT suppression might serve as protectant in DR. Moreover, *miR-29b* knockdown significantly reversed the effects of cell apoptosis induced by MIAT suppression, which indicated that the protective function of MIAT suppression was interfered by *miR-29b* knockdown.

In summary, our investigation identified a specific regulatory network of cell apoptosis that mediated by MIAT in DR. We revealed that the expression of MIAT was associated with NF-κB (p-p65), NF-κB activated the MIAT, MIAT target regulated *miR-29b* expression and finally regulated the cell apoptosis. Our present study showed that MIAT controlled the cell apoptosis in DR might be partly through absorbing *miR-29b* and inhibiting its function, meanwhile regulating the expression of Sp1. Further clinical therapy based on the NF-κB/MIAT/*miR-29b*/Sp1network appears to be important for DR.

## Abbreviations

Ad-GFP: adenovirus-carrying GFP
Ad-MIAT: adenovirus myocardial infarction associated transcript
DM: diabetic mellitus
DR: diabetic retinopathy
LncRNA: Long non-coding RNA
MIAT: myocardial infarction associated transcript
NC: negative control
NF-κB: nuclear factor κB
OD: Optical density
PI: propidium iodide
p-p65: phosphorylate-p65
rMC: retinal Müller cells
SPRI: solid phase reversible immobilization
STZ: streptozotocin
